# Global Intersection of Long Non-Coding RNAs with Processed and Unprocessed Pseudogenes in the Human Genome

**DOI:** 10.3389/fgene.2016.00026

**Published:** 2016-03-24

**Authors:** Michael J. Milligan, Erin Harvey, Albert Yu, Ashleigh L. Morgan, Daniela L. Smith, Eden Zhang, Jonathan Berengut, Jothini Sivananthan, Radhini Subramaniam, Aleksandra Skoric, Scott Collins, Caio Damski, Kevin V. Morris, Leonard Lipovich

**Affiliations:** ^1^Center for Molecular Medicine and Genetics, Wayne State UniversityDetroit, MI, USA; ^2^Department of Biotechnology and Biomedical Sciences, The University of New South WalesSydney, NSW, Australia; ^3^Department of Molecular and Experimental Medicine, The Scripps Research InstituteLa Jolla, CA, USA

**Keywords:** pseudogenes, lncRNA (long non-coding RNA), ESTs (expressed sequence tags), piRNA, gene expression, transcriptome, SNPs (single nucleotide polymorphisms), human disease

## Abstract

Pseudogenes are abundant in the human genome and had long been thought of purely as nonfunctional gene fossils. Recent observations point to a role for pseudogenes in regulating genes transcriptionally and post-transcriptionally in human cells. To computationally interrogate the network space of integrated pseudogene and long non-coding RNA regulation in the human transcriptome, we developed and implemented an algorithm to identify all long non-coding RNA (lncRNA) transcripts that overlap the genomic spans, and specifically the exons, of any human pseudogenes in either sense or antisense orientation. As inputs to our algorithm, we imported three public repositories of pseudogenes: GENCODE v17 (processed and unprocessed, Ensembl 72); Retroposed Pseudogenes V5 (processed only), and Yale Pseudo60 (processed and unprocessed, Ensembl 60); two public lncRNA catalogs: Broad Institute, GENCODE v17; NCBI annotated piRNAs; and NHGRI clinical variants. The data sets were retrieved from the UCSC Genome Database using the UCSC Table Browser. We identified 2277 loci containing exon-to-exon overlaps between pseudogenes, both processed and unprocessed, and long non-coding RNA genes. Of these loci we identified 1167 with Genbank EST and full-length cDNA support providing direct evidence of transcription on one or both strands with exon-to-exon overlaps. The analysis converged on 313 pseudogene-lncRNA exon-to-exon overlaps that were bidirectionally supported by both full-length cDNAs and ESTs. In the process of identifying transcribed pseudogenes, we generated a comprehensive, positionally non-redundant encyclopedia of human pseudogenes, drawing upon multiple, and formerly disparate public pseudogene repositories. Collectively, these observations suggest that pseudogenes are pervasively transcribed on both strands and are common drivers of gene regulation.

## Introduction

Non-protein-coding genes have emerged as prevalent functional elements in the human genome since the completion of the Human Genome Project. Highlighted by the ENCODE and FANTOM consortia (The ENCODE Project Consortium, 2012; FANTOM Consortium the RIKEN PMI CLST-DGT., 2014), these elements include tens of thousands of pseudogenes, as well as comparably numerous long non-coding RNA genes. Global patterns of pseudogene transcription and potential function are still poorly understood, but the field is of great importance for human disease due to the high sequence similarity between pseudogenes and their parental genes, which generates the potential for sequence-specific regulation. Recent case studies establish essential functional roles of both pseudogenes and lncRNAs in metazoan systems reviewed in Johnsson et al. (2014). A few of these studies have even highlighted the functional impacts of lncRNA transcription at pseudogene loci on the regulation of the pseudogenes' parental genes (Hawkins and Morris, 2010; Poliseno et al., 2010; Johnsson et al., 2014).

Antisense transcription makes three main regulatory mechanisms possible. The antisense transcript may hybridize to a gene promoter resulting in transcriptional regulation, transcripts may also be processed into short ncRNAs that bind to sense RNA transcripts from the same locus or from other loci, or these antisense transcripts may hybridize with sense transcripts that share high sequence similarity. This kind of hybridization leads to both positive and negative regulation of the sense transcripts in a context specific fashion (Katayama et al., 2005; Morris and Mattick, 2014). In this study, we identified all instances of pseudogene sense and antisense transcription, including specifically sense-antisense co-transcription of pseudogenes and lncRNAs from the same loci. We did this in order to compute the whole-transcriptome space of antisense-mediated pseudogene regulation, and by implication, to infer the impact of this regulation on the pseudogenes' parental genes.

## Methods

### Derivation of pseudogene-LncRNA overlaps from input data

To compute a comprehensive genome-wide potential network space of integrated pseudogene-lncRNA regulation, we developed and implemented an algorithm to identify all pseudogenes that are overlapped by lncRNA transcription in both sense and anti-sense orientations. Our algorithm imported three public repositories of pseudogenes: GENCODE v17 (all types of pseudogene transcripts; *n* = 15233); RetroAli5 from the UCSC Genome Database (unique processed transcripts; *n* = 13742) and Yale Pseudo60 (unique processed and unprocessed transcripts; *n* = 17216), plus two public lncRNA catalogs: Broad Institute (unique transcripts; *n* = 21537; Cabili et al., 2011) and GENCODE v17 (unique transcripts *n* = 22340). The file intersection hence comprised six pseudogene-lncRNA genomewide overlaps. We identified 2277 loci containing exon-exon overlaps between all pseudogenes and all lncRNAs (Supplementary Dataset 1). Of these, 1167 had some EST and full-length cDNA support, i.e., sense and/or antisense transcription of the lncRNA and/or the pseudogene, providing evidence of transcription (Supplementary Dataset 2). This project has generated an empirically supported transcriptome coverage matrix describing all pseudogene-lncRNA overlaps in the human genome. The resource serves as a foundation for future manual curation, parental-gene ontology analysis, and experimental validation of function of pseudogene-overlapping lncRNAs.

### Manual data annotation

In order to further characterize the transcribed human pseudogene dataset, we performed manual annotation of all loci in UCSC to confirm intersects, identify the pseudogenes' parental genes, and canvass NHGRI SNPs as well as piRNAs in the area. We also used the FANTOM Zenbu browser for CAGE validation to identify transcribed pseudogenes with >= 1 tpm (tags per million) values at regions which at least partially overlapped annotated piRNAs. NHGRI significant disease-associated SNPs were annotated when a disease associated SNP was either contained within a pseudogene's span, or resided within 10 KB of either end of the gene. In order to manually confirm the computationally inferred intersects of public transcriptome data with pseudogenes, UCSC Genome Browser was used with: all ESTs from GenBank, lncRNA transcripts from the Broad Institute, and human mRNAs from GenBank. Parental genes were also identified for use in pathway analysis using annotations contained within the pseudogene data sets, and NCBI BLAST results obtained on 4/19/2015-4/26/2015 from pseudogenes which lacked annotation. These results were then input into G:Profiler using default settings (Reimand et al., 2011), which identified over-expressed and under-expressed pathways in our 313 loci data set relative to pseudogenes taken from pseudogene.org's PsiCube database (Sisu et al., 2014). Additionally, the pseudogene.org parental gene database was compared against Ensemble 79 using the ontology tool G:Profiler, in order to identify ontological categories that are enriched or depleted amongst human pseudogenes' parental genes in the pseudogene.org database, when compared to all human genes.

### Data sets and the generation of an all-inclusive pseudogene database

Post-genomic biology has very recently identified a high prevalence of non-protein-coding functional elements in the human genome. Hence, the genome-wide significance as well as incidence of pseudogene-lncRNA overlaps is still poorly understood. To remedy this, we have performed global genome intersects of pseudogenes and lncRNA genes and evaluated empirical evidence of transcription at each pseudogene-lncRNA overlap using full-length cDNA (Genbank mRNA), EST, piRNA, and pseudogene support. For our purposes, we defined transcriptional support in our high confidence outputs as having both EST and full-length cDNA support. Nine different Gene Transfer Format (GTF)-formatted data sets were used. There were three compilations of pseudogenes used: GENCODE Version 17 Pseudogenes, Yale Pseudogenes 60, and UCSC's Retroposed Pseudogene Ali5. There were two lncRNA compilations used: GENCODE's Version 17 lncRNA table obtained from gencodegenes.org, and Broad Institute's LincRNA Transcripts taken from the UCSC Table Browser. The more inclusive category lncRNA (as opposed to lincRNA) was used for analysis because GENCODE's classification system defines an lncRNA gene (long non-coding RNA gene) as belonging to any one of several hierarchical categories. Those categories include lincRNAs (long intergenic ncRNAs, a small and well-defined subset of lncRNAs that reside completely outside of protein-coding gene loci); antisense lncRNAs (those that overlap protein-coding genes and that are therefore by definition not lincRNAs); and other lncRNA types. The evidence for these lncRNAs arises from cDNA and EST data with selective full-length RTPCR/RACE and sequencing validation (GENCODE), and from RNAseq (Broad). The GENCODE v17 pseudogenes were filtered to remove gene ID redundancy. An additional 2 data sets were used as indicators of expression. These data sets were obtained from the UCSC Table Browser. The first set was all human mRNAs (full-length cDNAs) from GenBank, and the second set was all human ESTs from GenBank. We reverted the stated orientation of all 3′ ESTs in order to correctly account for their biological direction of transcription. PiRNAs were identified using a data set constructed from the NCBI's database of known human piRNAs. A final dataset, used to identify clinical variants of possible relevance to phenotypes impacted by lncRNA-pseudogene overlaps, was NHGRI's GWAS Catalog, which contains NHGRI-curated significant disease-associated SNPs. It was obtained from the UCSC Table Browser. Datasets were processed using Active Perl v5.16.3.1603 obtained from activestate.com, and input files were all hg19-assembly-mapped GTF files.

### Computational inference of pseudogene-LncRNA intersects

**Figure 1:**
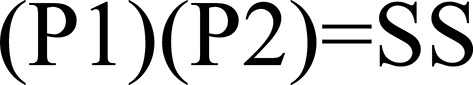
**Scoring formula for genomic-span overlaps**. P1, proportion of gene 1′s span overlapping with gene 2′s span; P2, proportion of gene 2′s span overlapping with gene 1′s span; SS, Span Span overlap score.

**Figure 2:**
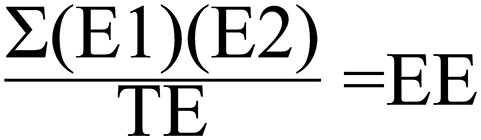
**Scoring formula for exon-to-exon overlaps**. E1, Proportion of Exon within Gene 1 overlapping with Exon within Gene 2. E2, Proportion of Exon within Gene 2 overlapping with Exon within Gene 1; TE, Total number of unique exons from both genes; EE, Exon Exon overlap score.

Six initial intersects were completed, with each pseudogene file being intersected with each lncRNA file. This process was done using gene IDs to define separate entries. Only gene entries from file 1 with genomic spans at least partially overlapping any of the genomic spans of entries from file 2 are marked as intersecting. Intersects are then scored to quantitatively represent how well the two spans and the two sets of exons' genomic coordinates match. Each matching Gene ID and score is then added to the additional information section with the format: (matching gene ID:span to span score:relative orientation:exon vs. exon score; Figures 1, 2). For subsequent intersects, which require exon-to-exon overlaps as opposed to merely genomic-span overlaps, only exons from file 1 which directly matched with an exon from file 2 are maintained.

### Scoring overlaps

The scoring method used for spans vs. spans and the one used for exons vs. exons yields a result ranging from 0 to 1. One represents two sets of genomic coordinates which are identical, while 0 represents no overlapping genomic coordinates whatsoever. The span vs. span score is calculated by multiplying the proportion of span number 1 which is covered by span number 2 by the proportion of span number 2 which is covered by span number 1 (Figure 1). The first step of exon vs. exon scoring is done by applying the above equation to each exon vs. exon overlap. The sum of the exon vs. exon scores generated from the first step is then divided by the total number of exon loci within gene 1 plus the number of exon loci within gene 2 which do not share genomic coordinates with any exon in gene 1 (Figure 2).

### Overlaps performed

Overlaps were performed to generate a final dataset containing pseudogenes supported by lncRNAs, full-length cDNAs, and ESTs for each initial pseudogene dataset (Table 1). These datasets were further analyzed by overlapping them with piRNAs and NHGRI GWAS SNPs. These intersections generated files for each pseudogene list, including one for each lncRNA intersect.

**Table 1:** **Computationally derived intersections of all pseudogene, lncRNA, cDNA, and EST databases**.

**File 1 GID**	**File 2 GID**	**Exon-exon gene IDs**	**Sense Overlaps**	**Antisense Overlaps**	**Complex Loci**
GENCODE 17 Pseudogenes	GENCODE 17 lncRNA	163	87	56	20
GENCODE 17 Pseudogenes vs. GENCODE 17 lncRNA	cDNA	64	33	4	27
GENCODE 17 Pseudogenes vs. GENCODE 17 lncRNA	EST	68	4	2	62
GENCODE 17 Pseudogenes vs. GENCODE 17 lncRNA	cDNA and EST	48	23	3	22
GENCODE 17 Pseudogenes	Human lincRNA	870	725	45	100
GENCODE 17 Pseudogenes vs. Human lincRNA	cDNA	371	216	15	140
GENCODE 17 Pseudogenes vs. Human lincRNA	EST	646	53	18	575
GENCODE 17 Pseudogenes vs. Human lincRNA	cDNA and EST	325	186	12	127
Retro Ali5	GENCODE 17 lncRNA	211	79	120	12
Retro Ali5 vs. GENCODE 17 lncRNA	cDNA	78	35	17	26
Retro Ali5 vs. GENCODE 17 lncRNA	EST	162	30	16	116
Retro Ali5 vs. GENCODE 17 lncRNA	cDNA and EST	69	32	15	22
Retro Ali5	Human lincRNA	557	405	108	44
Retro Ali5 vs. Human lincRNA	cDNA	129	61	26	42
Retro Ali5 vs. Human lincRNA	EST	381	64	34	283
Retro Ali5 vs. Human lincRNA	cDNA and EST	113	53	23	37
Yale 60	GENCODE 17 lncRNA	105	66	25	14
Yale 60 vs. GENCODE 17 lncRNA	cDNA	40	25	2	13
Yale 60 vs. GENCODE 17 lncRNA	EST	46	2	1	43
Yale 60 vs. GENCODE 17 lncRNA	cDNA and EST	31	18	1	12
Yale 60	Human lincRNA	547	468	24	55
Yale 60 vs. Human lincRNA	cDNA	192	123	6	63
Yale 60 vs. Human lincRNA	EST	372	38	12	322
Yale 60 vs. Human lincRNA	cDNA and EST	157	96	5	56

*Directionality is relative to pseudogene locus, and complex loci refer to intersects which contain both sense and antisense overlaps*.

### Output data format for pseudogene-LncRNA overlap computations

The outputs of these intersects are in a GTF format, with a single line devoted to each non-overlapping unique pseudogene Gene ID. Each pseudogene gene ID entry row contains all of its original information, but also includes all gene and transcript Ids from intersected data sets. The lncRNA gene IDs which intersect with the original pseudogene ID each have relative strandedness (i.e., sense or antisense relative to the pseudogene) and quality of overlap information (quantitatively score at both genomic-span and exon levels as summarized above) added to their entries.

#### Redundancy elimination

**Figure 3:**
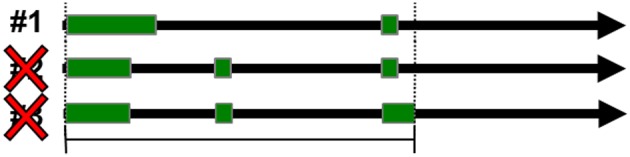
**Positional redundancy elimination**. Redundant loci were identified by overlapping genomic spans and were eliminated so that only one locus could occupy the same genomic span.

Redundancy within the data sets was controlled for by removing entries that shared gene and transcript ID's. Redundancy of loci was also controlled for by removing all but the first entry of overlapping genomic spans when spans were mapped to hg19 (Figure 3). The first entry in the GTF file corresponds to the transcript with the lowest starting genomic coordinate.

## Results and discussion

### Evidence supporting pseudogene expression in public transcriptome and annotation datasets

We define public transcriptome datasets as EST and full-length cDNA data in Genbank. We define lncRNA annotation support as the Broad Institute lincRNA and GENCODE lncRNA datasets and their corresponding tables in the UCSC Genome Browser. A total of 696 unique multiple-block pseudogene IDs had both public transcriptome support and lncRNA annotation support. This set of 696 gene IDs was collapsed to 439 unique loci as defined by non-overlapping genomic coordinates. Some of these loci had single-strand transcription (either sense or antisense relative to the pseudogene, but not both) and some had dual-strand transcription as defined by the transcriptome or annotation data. An additional 1448 single-block pseudogene loci were identified, with 728 unique single-block pseudogene loci. The combination of these two sets of pseudogenes results in a total set of 1167 unique high-confidence pseudogene loci. Next, we deployed two different criteria for discovery of unique loci containing pseudogene-lncRNA overlaps on one or both genomic strands. The rationale was to gauge both the accuracy and the transcriptomic representation of the GENCODE and Broad lncRNA datasets, by determining the extent to which the exons of these lncRNAs are supported by independent public transcriptome data. The 1167 unique loci were derived from cases where EST and mRNA support is present, but not necessarily bidirectional, at each locus. The more stringent criterion of bidirectional EST and mRNA support at each locus required EST and full-length cDNA support on both sense and antisense strands, with at least a partial exon-to-exon overlap of the sense and the antisense transcripts. The biological basis for this criterion is given by the potential of spliced, processed sense-antisense RNA hybrids (Katayama et al., 2005; Johnsson et al., 2013) to exert post-transcriptional, including cytoplasmic, regulation, in contrast to the purely epigenetic regulatory modalities conferred by unprocessed nuclear sense-antisense hybrids (Vadaie and Morris, 2013). Imposing this criterion yielded a group of 313 loci that is a subset of the 1167 high-confidence loci. This group of 313 loci was utilized as the minimum, most conservative dataset for manual annotation. Earlier studies have indicated that sense and antisense lncRNAs from pseudogene loci have distinct roles in parental gene regulation (Hawkins and Morris, 2010; Johnsson et al., 2013). Motivated by this finding, we carried out manual annotation on this entire dataset, in order to derive a genome-wide functional understanding of the lncRNA pseudogene relationship, including gene ontologies, and the extent to which the parental genes of these pseudogenes may participate in common functional pathways.

### Evidence for PiRNA biogenesis from regions of non-coding pseudogene transcription

There is limited evidence in the literature for short RNA biogenesis from mammalian sense antisense overlaps (Werner et al., 2014). Accordingly, we tested the hypothesis that bidirectional transcription at pseudogene-lncRNA overlap regions may generate short non-coding RNAs from long precursors. We defined piRNAs using the nucleotide division of GenBank (NCBI) to select only sequences identified as piRNAs in humans. Having constructed a custom database of 44264 piRNAs, we found that 367 of the 1167 loci have unidirectional or bidirectional piRNA support at one or more of the exons that were also supported by both public transcriptome data and lncRNA annotation data. These 367 loci contained an average of 61 piRNA gene IDs per locus. Of the 313 loci which exhibited bidirectional transcription support in both full-length cDNAs and EST data, 109 shared at least one exonic base with a piRNA. The data set of 1167 loci has 31.45% loci support by piRNA, while the conservative data set of 313 bidirectionally-transcribed exon-to-exon lncRNA-pseudogene overlaps showed a 34.82% loci support by piRNA. The Z-Score comparing these two data sets using a one tailed test is −1.1355. The corresponding *p* = 0.12714, providing insufficient evidence to conclude that there is enrichment of piRNAs in the 313 loci data set vs. the 1167 loci data set.

### Bidirectionally transcribed loci allow for multiple pseudogene regulatory mechanisms

Because ESTs are more numerous than full-length cDNAs, they are useful as a window into a part of the transcriptome that may not have been adequately sampled by full-length clones in public databases. Accordingly, we analyzed the prevalence of sense, antisense, and bidirectional exon-exon overlap support across full-length cDNA and EST data using the 1167 pseudogene dataset, which required supporting lncRNA exon-exon overlaps with each pseudogene. The result was that in full-length cDNA data alone, most loci had sense only full-length cDNA support, followed by sense antisense support, and antisense only full-length cDNA support, respectively (Table 2). Concordant with increased overall EST prevalence, bidirectional EST support was more common, whereas unidirectional EST support followed a similar sense to antisense ratio as that of full-length cDNA support. Summarily, this points to sense-strand transcription of pseudogenes being more prevalent than bidirectional or antisense transcription.

**Table 2:** **Directionality of lncRNA-supported pseudogene loci relative to the direction of pseudogene transcription in cDNA and EST data**.

**Support from**	**Bidirectional**	**Antisense only**	**Sense only**
cDNA	374	164	629
EST	836	88	243
EST and cDNA	313	53	196

The 313 loci comprise a high-confidence dataset of lncRNA-pseudogene intersections because exon-to-exon sense and antisense overlap was confirmed in both full-length cDNA and EST data for each locus. These loci represent subsets of two gene categorizations which are poorly conserved outside of the human genome. Statistical evidence supports conservation in a small set of pseudogenes, but many are the result of recent insertions (Ohshima et al., 2003; Svensson et al., 2006). While, 60% of human lncRNAs lack conservation outside of primates (Necsulea et al., 2014; Washietl et al., 2014). This raises the possibility that each of these loci may impact complex regulatory networks through multiple mechanisms dependent on and made possible by the empirical co-existence of sense and antisense transcription. Published evidence supports the contention that sense-antisense co-transcription is a powerful regulatory mechanism in mammalian systems (see Section Introduction). Because our loci contain sense-antisense pseudogene-lncRNA overlaps, the pseudogenes may act in cis on cognate (antisense) lncRNA target genes, and not only in trans on their parental genes as previous literature suggests. The in-cis effects on non-parental genes (genes in the vicinity of each sense-antisense overlap, casually unrelated to the parental genes of the pseudogene which are located distantly or on other chromosomes) could conceivably be generated by mechanisms unrelated to the pseudogene itself, such as expressed repetitive sequences in the exons of the lncRNAs. Additionally, chromatin modifications spreading from the pseudogene, and affecting large genomic regions could also generate a cis-effect (Milligan and Lipovich, 2014).

### Manual validation of computationally inferred pseudogene-LncRNA intersects

In order to validate the accuracy of the pseudogene-lncRNA exon-to-exon overlaps identified by our program, we carried out manual annotation of all 313 loci in the UCSC Genome Browser (Kent et al., 2002). In a few cases, manual annotation failed to garner support for our computational pipeline-inferred evidence for pseudogene expression. Those cases were due to ESTs from a particular set of random-primed libraries that later turned out to be contaminated with unspliced, singleton, and genomic-DNA products (Dias Neto et al., 2000) and RAGE ESTs corresponding to initiation events from artificially introduced promoters unrelated to any endogenous gene expression (Harrington et al., 2001), two problematic datasets that had been included in the Genbank set of ESTs we had used for computational identification of expression. ORESTES ESTs are unreliable due to a high proportion of singleton un-spliced ESTs, and RAGE ESTs are derived from cell lines utilizing artificial promoters, making them unreliable measures of expression. However, in the vast majority of cases, our UCSC Genome Browser-assisted manual annotation confirmed the computationally inferred sense-antisense overlaps of lncRNAs and pseudogenes, thereby providing prevalent validation of our pipeline's performance.

When intersecting the 313 loci with the NHGRI GWAS catalog, 7 loci with NHGRI GWAS significant disease-associated SNPs either within the loci or within 10 kb of the loci were revealed. A subset of three NHGRI GWAS SNPs resided entirely within the bidirectionally transcribed exon-to-exon lncRNA-pseudogene overlap loci. The three polymorphisms were: rs1805007, rs8103033, rs13017599. The rs1805007 polymorphism is contained within the MC1R gene locus and is associated with an increased risk of skin cancer (Zhang et al., 2013). The rs8103033 polymorphism is contained within the LGALS17A gene locus and is associated with an increased risk of childhood obesity (Hindorff et al., 2009). The rs13017599 polymorphism is 5600 bases downstream of the 3′ end of the REL gene, and is associated with an increased risk of rheumatoid and psoriatic arthritis (Ellinghaus et al., 2012). We hypothesized that cleavage of longer RNAs at sense-antisense loci, followed potentially by 5′-end recapping of the cleaved RNA biogenesis products, may be a prerequisite to short RNA, such as piRNA, biogenesis. Therefore, we examined CAGE data for evidence of recapped RNA processing intermediates on both strands at each locus. However, amongst our piRNA-containing loci, we saw evidence of cleavage and recapping at only one locus (PGOHUM00000251074) (Supplementary Figure 1). The parental gene of the PGOHUM00000251074 pseudogene is PARP8, a poly ADP-ribose polymerase gene with high mRNA abundance in multiple tissues.

### Parental gene ontology

**Figure 4:**
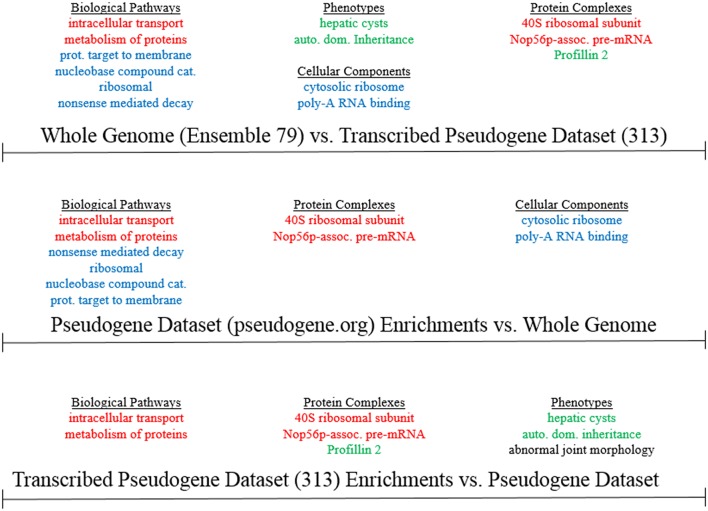
**Gene Ontology Comparison of all human protein-coding genes Ensembl 79, the PsiCube pseudogene parental gene database (from pseudogene.org), and the parental genes of the 313 loci computationally identified as bidirectionally-transcribed lncRNA-overlapping pseudogenes**. Enrichments which are common to the 313 loci vs. both the whole genome and the parental gene database are in green. Enrichments found in all three comparisons are in red, and those which are unique to either pseudogene parental gene database when compared with the whole genome are in blue.

Using the online G:Profiler tool (Reimand et al., 2011), we computed gene ontologies for the 231 unique parental gene loci from the 313 loci expressed pseudogene list. We set out to interrogate whether specific functional categories, pathways, processes, or disease associations are enriched, or depleted, in the set of human pseudogenes characterized by exon-to-exon lncRNA overlaps and bidirectional transcription, relative to the entire known space of human pseudogenes. As a representative distribution of all pseudogenes' parental gene functions, we used the psiCube genomewide database of human pseudogenes' parental genes at Pseudogene.org (Sisu et al., 2014). In order to compute significant ontological results, we used the g:SCS (Set Counts and Sizes) algorithm, which corrects raw *p*-values for use in gene ontology calculations, and is discrete from other multiple testing corrections in that it assumes that genes associated with a specific term are also associated with all more general parental terms. Our results showed significant enrichments, but not depletions, for specific ontological categories at a *p* = 0.05 threshold in the comparison of the parental genes of our 231 (313) pseudogenes to the whole-genome parental-gene list (Figure 4). When comparing the transcribed pseudogene list to the whole human reference genome, the most significantly enriched biological pathways were nonsense mediated RNA decay, ribosomal components, and protein ER localization. However, these same pathways were also identified as enriched in the pseudogene.org parental gene dataset relative to the whole-human-genome list of all protein-coding genes (which includes genes without pseudogenes, as well as parental genes of pseudogenes). This could indicate that these pathways are genuinely enriched in our transcribed pseudogene list, or that these pathways are generally enriched in all pseudogenes, regardless of their lncRNA overlaps or their transcription (Supplementary Figure 2). In contrast, when comparing our bidirectionally-transcribed lncRNA-overlapping pseudogene list to the list of parental genes of all human pseudogenes, there was significant enrichment in autosomal dominant hereditary diseases, genes relevant to hepatic cysts, abnormal joint morphology, and Profilin 2 Complex genes (Supplementary Figure 3). These same enrichments, except for abnormal joint morphology, were also observed when comparing the 231 unique parental genes of our 313 transcribed pseudogenes to the reference human genome gene lists. Based on the datasets available to us, it is therefore possible that our transcribed pseudogene list may be truly enriched in these pathways, and that these pathways are biologically associated with pseudogene-lncRNA exonic overlaps and with sense-antisense co-transcription of these overlaps.

### A comprehensive non-redundant human pseudogene catalog

**Figure 5:**
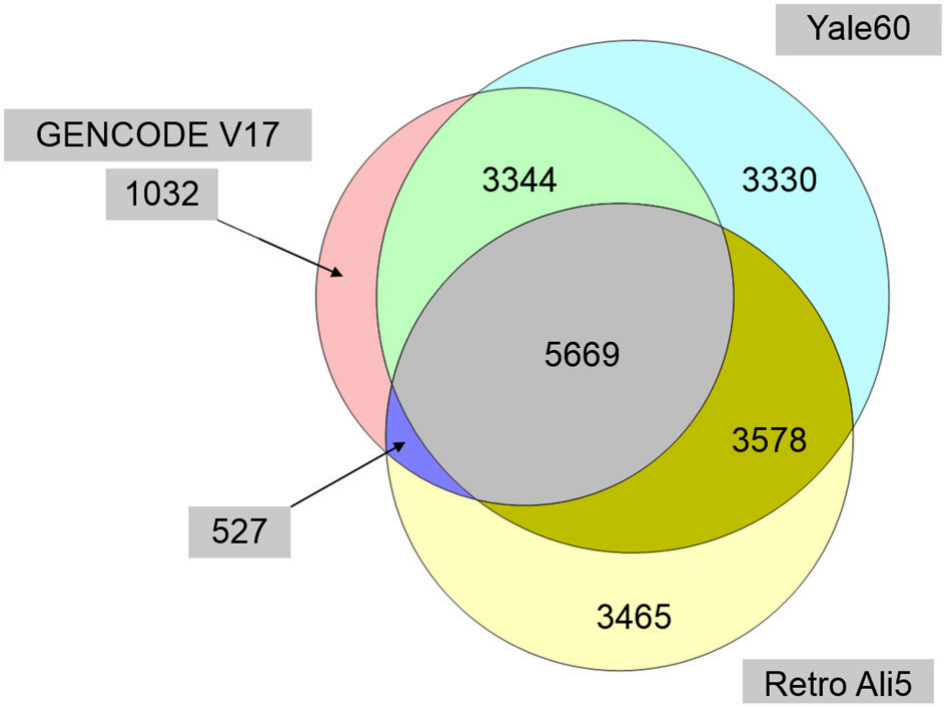
**Venn diagram of the genomic positionally-nonredundant intersection of three major public pseudogene databases**. The resulting non-redundant dataset (Supplementary Dataset 3) renders a more inclusive and comprehensive pseudogene database of 20945 pseudogene loci and alleviates problems due to accession number synonymity within and between the three databases. (Accession number synonyms point to the same pseudogene along the human genome, but in the absence of positionally-nonredundant collapsing, they may be misrepresented by downstream programs as representing multiple pseudogenes).

In the process of cataloging pseudogene-lncRNA overlaps, we realized that a genome-wide, positionally non-redundant overlap of the several currently available public pseudogene collections had not yet been performed. The GENCODE pseudogene resource (psiDR) is itself an integration of pseudogene annotation data from Pseudopipe, retroFinder, HaVANA, ENCODE, and the 1000 Genomes Project (Pei et al., 2012). This resource provides a high confidence but fairly exclusive dataset which utilizes manual annotation, computational gene prediction, and functional annotation data. We found this resource to be too conservative, and sought to facilitate future studies requiring a single positionally non-redundant catalog of unique pseudogene loci by merging data from three datasets to derive a more inclusive superset of data. We used data from the Yale 60, GENCODE V17, and UCSC Retro-Ali5 pseudogene datasets to compile a non-redundant database of all unique pseudogene loci mapped to hg19 (Figure 5). This final dataset contains 20945 non-overlapping pseudogene loci, greatly expanding upon the number of non-redundant loci found in any one of the three original datasets (e.g., 10562 in GENCODE V17, 15921 in Yale60, and 13239 in Retro Ali5; Supplementary Dataset 3). The introduction of a new and more comprehensive dataset will facilitate *in-silico* discovery of functional pseudogenes, because the dataset can function as a standardized repository that is not compromised by the positional redundancy of multiple pseudogene accession numbers mapping to the same locus and representing the same single actual pseudogene. The discovery of these functional pseudogenes may allow for a better understanding of future GWAS datasets, because significant disease-associated SNPs can now be systematically overlapped with the promoters and the exons of non-redundant pseudogenes.

## Conclusions

We have non-redundantly integrated pseudogenes from three public sources, arriving at approximately 20945 non-redundant pseudogene loci, creating an invaluable foundation for regulatory genomic analysis of pseudogene function. This integrated pseudogene library, combined with our transcribed pseudogene dataset, suggests that at least 5% (1167 of 20945) of human pseudogenes are transcribed in either sense or antisense orientation. This percentage may represent a lower bound, because out initial screen constrained the expression search of full-length cDNA and EST data only to pseudogenes that had at least a partial genomic-span overlap with lncRNAs, as opposed to all human pseudogenes. Further, among the 1167 pseudogene loci we identified as transcribed, 313 high-confidence loci exhibit exon-to-exon overlaps between annotated pseudogenes and annotated lncRNAs, with full-length cDNA and EST support for bidirectionality of transcription at these overlaps. These events suggest the potential for epigenetic as well as post-transcriptional regulation of pseudogenes by lncRNAs, of lncRNAs by pseudogenes, and of the pseudogenes' parental genes by the lncRNA-pseudogene overlaps, while fitting the definition of complex loci (Engström et al., 2006). Therefore, bidirectional transcription is robust at pseudogene-lncRNA intersections, although further work is necessary to estimate the extent to which sense-antisense pseudogene-lncRNA co-transcription occurs in the same tissues or in the same single cells. Gene ontology analysis of this data subset suggests that bidirectionally transcribed lncRNA-pseudogene overlaps exhibit functional category enrichments that are not mirrored in comparisons of all pseudogenes' parental genes to all human genes, and that these enrichments may underlie specific human diseases. Our work also demonstrates the continuing utility of unambiguously-mapped, full-length transcriptome data (Genbank cDNAs) for the study of complex loci; due to the inherent ambiguity of short-tag (e.g., second-generation RNAseq such as Illumina) mappings to transcribed pseudogenes, further full-length analyses of the lncRNA-pseudogene transcriptome intersection with emerging third-generation technologies (e.g., Pacific Biosciences) are warranted. The 313 loci generated in this study will be a useful tool when looking at gene regulatory networks.

## Author contributions

MM was responsible for: writing the majority of the article, coordinating the review, writing the code for the programmatic analysis, validating manual annotation, gene ontology work, making the figures. LL was responsible for: writing a significant part of the article, reviewing the finished work, guiding the research, providing the funding for the research, coordinating with other authors, and directing the course in which manual annotation was performed. KM was responsible for: writing a significant part of the article, reviewing the finished work, coordinating with other authors, and directing the course in which manual annotation was performed. EH: a student in the undergraduate course that contributed extra work in addition to participating in the original manual annotation of the loci, participated in reviewing the finished work. AY, CD: students in the undergraduate course that contributed extra work in addition to participating in the original manual annotation of the loci. AM, DS, EZ, JS, JB, RS, AS, and SC: were students in the undergraduate course that participated in the manual annotation of the loci.

## Funding

The National Institute of Health's 1DP2-CA196375 award supported the salary of MM as a part-time research faculty during the time-period in which this work was completed.

### Conflict of interest statement

The authors declare that the research was conducted in the absence of any commercial or financial relationships that could be construed as a potential conflict of interest.
