# Supplementary figures and images for: Global Intersection of Long Non-Coding RNAs with Processed and Unprocessed Pseudogenes in the Human Genome
Source: Front Genet. 2016 Mar 24;7:26. doi: 10.3389/fgene.2016.00026 (PMC4805607; doi:10.3389/fgene.2016.00026)

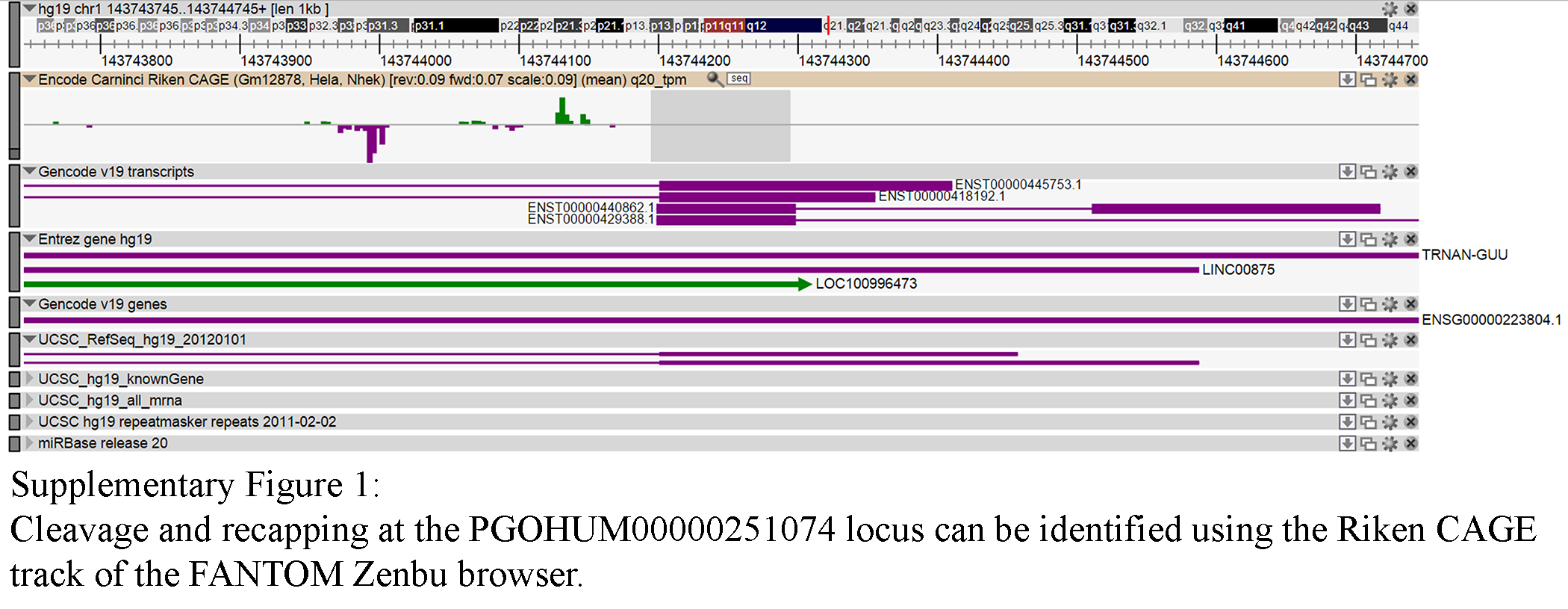

Supplement: Supplementary file 4 [file Image1.TIF]

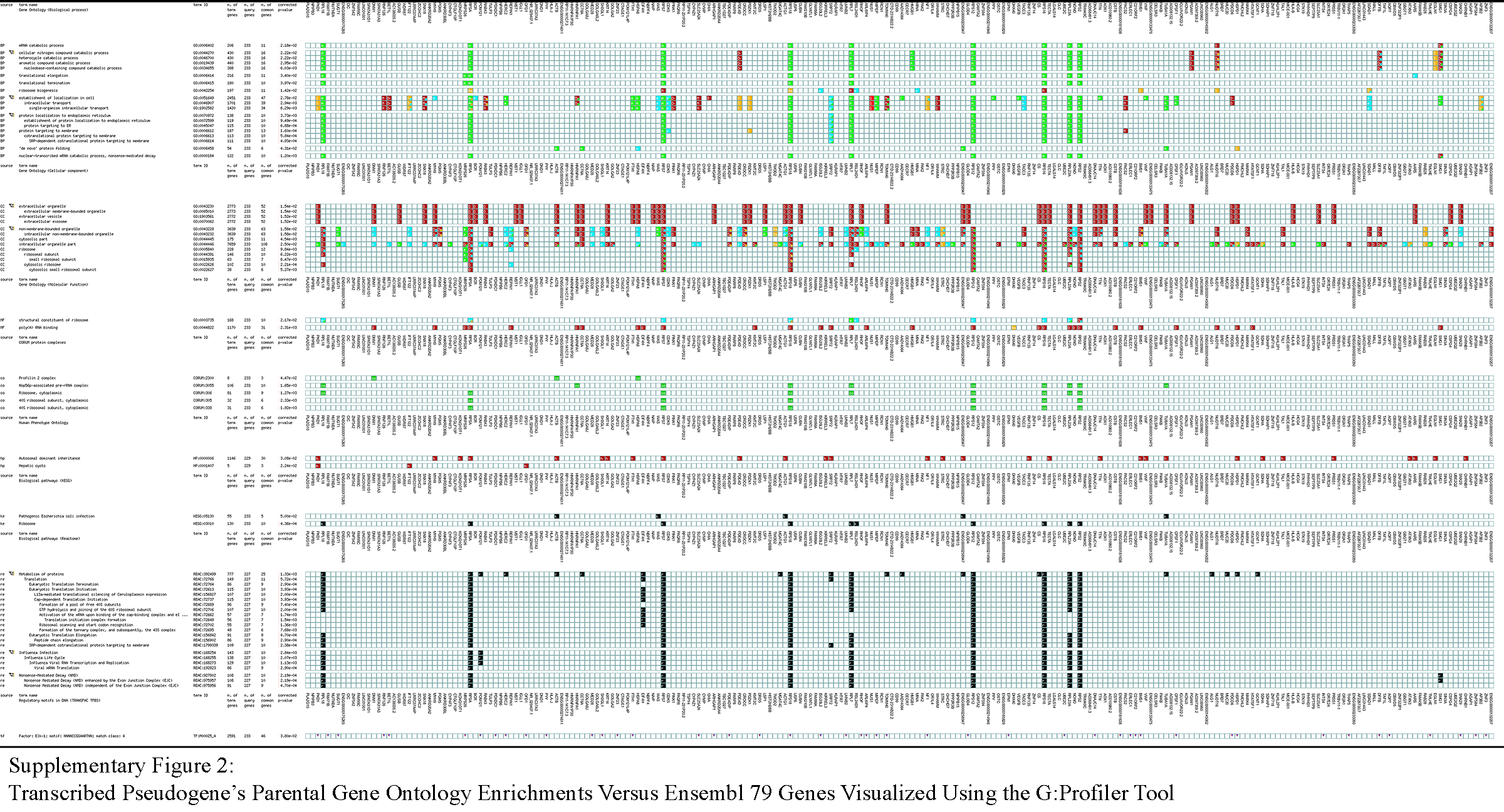

Supplement: Supplementary file 5 [file Image2.TIF]

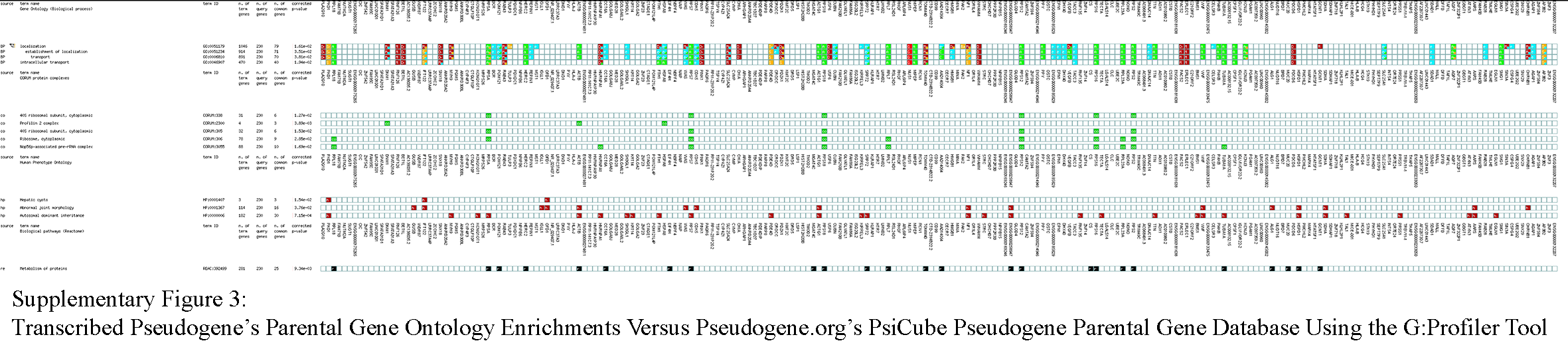

Supplement: Supplementary file 6 [file Image3.TIF]
